# Use of Repeated Measures Data Analysis for Field Trials with Annual and Perennial Crops

**DOI:** 10.3390/plants11131783

**Published:** 2022-07-05

**Authors:** Paulo Pagliari, Fernando Shintate Galindo, Jeffrey Strock, Carl Rosen

**Affiliations:** 1 Department of Soil, Water, and Climate, Southwest Research and Outreach Center, University of Minnesota, Lamberton, MN 56152, USA; jstrock@umn.edu; 2 Center for Nuclear Energy in Agriculture, University of São Paulo, Piracicaba 13416-000, SP, Brazil; fsgalindo@usp.br; 3 Department of Soil, Water, and Climate, University of Minnesota, Twin Cities Campus, St. Paul, MN 55108, USA; crosen@umn.edu

**Keywords:** statistical analysis, repeated measures, agronomic field trials, random effects, in season sampling, ANOVA, covariance structure

## Abstract

Field studies conducted over time to collect any type of plant response to a set of treatments are often not treated as repeated measures data. The most used approaches for statistical analyses of this type of longitudinal data are based on separate analyses such as ANOVA, regression, or time contrasts. In many instances, during the review of manuscripts, reviewers have asked researchers to treat year, for example, as a random effect and ignore the interactions between year and other main effects. One drawback of this approach is that the correlation between measurements taken on the same subject over time is ignored. Here, we show that avoiding the covariance between measurements can induce erroneous (e.g., no differences reported when they exist, or differences reported when they actually do not exist) inference of treatment effects. Another issue that has received little attention for statistical inference of multi-year field experiments is the combination of fixed, random, and repeated measurement effects in the same statistical model. This type of analysis requires a more in-depth understanding of modeling error terms and how the statistical software used translates the statistical language of the given command into mathematical computations. Ignoring possible significant interactions among repeated, fixed, and random effects might lead to an erroneous interpretation of the data set. In this manuscript, we use data from two field experiments that were repeated during two and three consecutive years on the same plots to illustrate different modeling strategies and graphical tools with an emphasis on the use of mixed modeling techniques with repeated measures.

## 1. Introduction

Experiments in which successive measurements of a response variable on each of *n* experimental units or subjects (person, plots, plants, animals, etc.) taken over time are defined as *repeated measures* experiments [1,2]. For experiments conducted under field conditions, the subject term is usually the individual plot. Classic examples of repeated measures include weekly measurements of animal weight or plant height and diameter thickness, with the objectives of establishing growth curves, or hourly measurements of patient response to a certain drug or group of drugs to establish response curves. In these cases, the period of time (hours, weeks, and, in certain cases, months) between successive observations is relatively short. When longer time lags (e.g., a year) are required to measure subject response to a set of treatments, for example, in agronomic research where an experiment is re-started each growing season, measurements taken on the same subject (plots) are assumed to be independent and not correlated. As a result, repeated measures techniques are often not used. In cases similar to those just described, the covariance between measurements taken at different times on the same subject is disregarded, and consequently, error terms can be inflated, and statistical inference can be invalidated. Hence, the following question can be asked: Is it appropriate to treat yield and other measurements taken on annual and perennial plants in response to the same treatments, taken over successive years on the same plots, as a repeated measure experiment? 

Littell et al. [3] presented univariate and multivariate methods for analyzing repeated measures data, including analyses of time contrast variables and separate analyses at each point in time. Neither univariate nor multivariate methods take into consideration the correlation structure of the longitudinal setting (from one measurement to the next on the same subject, which can be time or distance). In contrast, the mixed modeling approach is the most flexible method in terms of handling the covariance among repeated measures. However, there are many considerations that need attention when adding a correlation structure to a statistical model, and selecting the most appropriate correlation structure with each specific data set is important. This issue is discussed in more depth further in this manuscript.

Taking a different approach, Loughin [4] and Loughin et al. [5] suggested the use of the staggered-start design for experiments with variables measured at different times over different years on the same plots. The variables, time and year, are confounding factors in statistical models of trials that are not staggered at the start of the trial. The staggered-start approach can, very efficiently, separate all of the random variation that is due to the “year” from the variation that is due to the “time” effects. There is also a series of other considerations about the staggered-start design that needs to be addressed from an agronomic and practical standpoint. The first and most important is that the majority of studies conducted under field conditions are funded for a relatively short period of time, and the researcher might not have the time and/or funding to stagger the proposed study; second, most of the research performed by graduate students can, usually, only be replicated for two to four years without significant increases in graduate school costs for the adviser and in time to pursue the degree; finally, experiments conducted under field conditions often include information about specific effects of weather on the treatments being tested. Being able to understand how seasons drier or wetter than long-term averages can affect the expected treatment response is important from an agronomic standpoint. By using the staggered-start design as presented by Loughin [5], one might not be able to address all of the concerns presented. 

Because the use of the staggered-start design is often not a viable option for most short-term agronomic field trials, alternative statistical analyses are needed when data sets are collected on the same plots over a few years. From here on, we deal with statistical approaches that might be more appropriate for experiments conducted under field conditions replicated over years. The reader is referred to Loughin and Loughin et al. [4,5] for more details on the staggered-start design. This manuscript is organized into several different topics, starting with the methodology and followed by applications. However, we tried to write the paper in a way that allows the reader to skip in and out of the sequence and yet be able to follow each topic alone.

## 2. Importance of Modeling Correlation and Covariance Components in a Model

When experiments are repeated over time, the researcher often expects that the treatment with the highest overall mean will have the highest mean at all sampling times, which would make the observations correlated, with a correlation (*ρ*). However, in most, if not all, field trials, the covariance (*σ*) within subjects will vary at each time (hereinafter, the effect of time is abbreviated as *t*) at which the measurements were taken, usually due to the random variation in the field during each year. The assumption that this *σ* is not present can result in the calculation of incorrect statistical tests. That is because the covariance term is calculated from the residuals after all variation due to the fixed effects in the model has been explained [6]. By removing the within-subject variance from the residuals, we can estimate more precise error terms used in *F*− and *t*-tests. This is illustrated later in the manuscript when we perform statistical analysis on a corn (*Zea mays*) data set.

There are several mathematical methods that can be used to estimate covariance terms [6,7]. However, it is important to model the best covariance structure for each data set, as its estimation will have a direct effect on the estimation of the experimental error term and standard error terms used in the *F*− and *t*-tests. The simplest covariance model assumes homogeneous variance (*σ*^2^) at all times, Var[*Y_ijt_*] = *σ*^2^ (*residual variance*) for all *t*, and also assumes that *σ* and *ρ* are 0. This model is referred to as Independent [6] and is the covariance model automatically used when year is treated as a fixed effect, whether the researcher knows it or not.

A slightly more complex covariance model assumes homogeneous variances at all times, Var[*Y_ijt_*] = *σ*^2^ for all *t*, and also homogeneous *σ* for all observations made on the same subject (hereinafter, each subject is abbreviated as *i* on the level of treatment and *j* on the level of plot), Cov[*Y_ijt_, Y_i’j’t’_*] = *σ_tt’_* = *ρσ*^2^, for all *i* = *i’* and *j* = *j’*. This model calculates only two parameters, *σ*^2^ and *σ_tt’_*, which is called Compound Symmetry [6], and in SAS^®^, it is specified as CS in the “TYPE = CS” option. Furthermore, we can see from the term Cov[*Y_ijt_, Y_i’j’t’_*] = *σ_tt’_* = *ρσ*^2^ that as *ρ* gets close to 1, the estimate of *σ_tt’_* will get closer to the estimate of *σ*^2^, but as *ρ* approaches 0, the estimate of *σ_tt’_* will also come close to 0. Therefore, when *ρ* is irrelevant (*ρ* = 0), the addition of a correlation component to the model is less likely to affect the statistical analysis. However, when there is a relevant *ρ* (*ρ* ≠ 0), the proper statistical approach for inference on treatment effects must be used. This model is formally equivalent to a simple split-plot specification if year is interpreted as a (whole-plot) grouping factor. For example, each block of four plots is viewed as a whole plot, and each plot is a split-plot with the treatments being applied at the split-plot level. It is important to note that this is just a formal identification since, in a split-plot design, both factors (year and treatment) are randomized: year at the whole-plot level and treatment at the split-plot level. This is not the case in a repeated measures structure, where year cannot be randomized. 

The most complex covariance model assumes heterogeneous *σ*^2^ at all times (*σ*^2^*_t_*, …, *σ*^2^*_t’_*) and heterogeneous *σ_tt’_* between any two observations taken on the same subject at times *t* and *t’*. In other words, this model calculates one *σ* for each pair of observations; for example, Cov[*Y*_*i*1_, *Y*_*i*’2_] = *σ*_12_, for all *i* = *i’*, is the covariance between time 1 and time 2; Cov[*Y*_*i*1_, *Y*_*i*3_] *σ*_13_, is the covariance between time 1 and time 3, …; and *σ_tt’_*, is the covariance between time *t* and time *t’*. Here, it is easy to see that this model is very complex and calculates a high number of covariance parameters [*t* (*t* + 1)/2]. This model is called Unstructured and, in SAS, is specified as UN. 

In most repeated measures experiments, the covariance is expected to decrease as the lag between times *t* and *t’* increases [8]. This is because the covariance term is based on the correlation between measurements taken at times *t* and *t’* on the same subject. This correlation is more likely to decrease with an increase in the time lag between measurements. An example of a covariance structure that decreases with time is the first-order autoregressive [AR (1)] (Littell et al., 2000). This model assumes homogeneous variances at all times, Var[*Y_ijt_*] = *σ*^2^ for all *t*, but assumes heterogeneous *σ* between any two observations taken on the same subject at times *t* and *t’*, Cov[*Y_ijt_, Y_i’j’t’_*] = *σ_tt’_* = *ρ^t’-t^σ*^2^, for all *i* = *i’*, *j* = *j’*, and *t* ≠ *t’*. The term *ρ^t’-t^* shows how the covariance decreases with an increase in the lag between measurements. For example, the covariance between years 1 and 2 will have the form of *ρ*^2−1^ *σ*^2^ = *ρσ*^2^; the covariance between years 1 and 4 will have the form of *ρ*^4−1^*σ*^2^ = *ρ*^3^*σ*^2^; and the covariance between years 3 and 4 will have the form of *ρ*^4−3^*σ*^2^ = *ρσ*^2^. Here, we see that this model calculates only two parameters, *ρ* and *σ*^2^. 

The different ways of calculating the covariance show the importance of modeling the most appropriate structure. We demonstrate throughout the paper how the addition of a covariance structure affects statistical analysis and, therefore, the importance of selecting the most appropriate one. 

## 3. Building *t*-Tests

It is also important to understand how the addition of correlation may alter the comparison between treatment means. For example, assuming homogeneous variances between year one and year two and a covariance of 0, the standard error used to test treatment differences between and within subjects (*Y_ijt_ − Y_ijt’_*) is calculated using model 1:

*√* [2 * (*σ*^2^)/(*r*)] (1)

where *√* represents the square root, *σ*^2^ is the pooled residual term variance, and *r* is the number of replicates. 

When the variances are heterogeneous among times, the error term to test differences between subjects at a given time *t* (*Y_it_ − Y_i’t_*) becomes model 2:

*√* [2 * (*σ*^2^*_t_*)/(*r*)] (2)

where *σ*^2^*_t_* is the residual term variance at time *t*, and *r* is the number of replicates. The error term to test differences within subjects at time *t* and *t’* given a treatment *i* (*Y_it_ − Y_it’_*), such that *i* = *i’* for a data set with heterogeneous variances is model 3:

√ {2 * [(*σ*^2^*_t_* + *σ*^2^*_t’_*)/2]/*r*} (3)

where *σ*^2^*_t_* is the residual term variance at time *t*; *σ*^2^*_t’_* is the residual term variance at time *t’*; and *r* is the number of replicates.

From these examples, Models (1)–(3) do not use correlation to build standard errors to estimate *t*-tests. However, if we assume that the variance between subjects is ≠ 0, we add a correlation between any two observations made on the same subject (*Y_ij_*). This allows us to account for variability that is due to the within-subject effect measured at times *t* and *t’*. For example, the standard error used to test the treatment effects at any time (*Y_ijt_ − Y_i’jt’_*) for all *i* ≠ *i’*, considering a model with homogeneous variances and homogeneous correlation (e.g., Compound Symmetry, CS), is model 4:

*√* [2 * (*σ*^2^*_CS_*)/(*r*)] (4)

where *σ*^2^*_CS_* is the residual term variance, and *r* is the number of replicates. The subscript (cs) is used to differentiate Equations (1) and (4), as they look the same, but their calculations yield different results due to the added correlation. In addition, this model shows that observations taken on different treatments in any year are independent. 

The error term to test differences within subjects at times *t* and *t’* given treatment *i* = *i’* (*Y_ijt_ − Y_i’jt’_*) is model 5:

*√* {2 * [(*σ*^2^*_CS_*/*r*) − (*σ_tt’_*/*r*)]} (5)

where *σ*^2^ is the variance, *σ_tt’_* is the covariance for measurements taken on subject *j* assigned to treatment *i* at times *t* and *t’*, and *r* is the number of replicates. This model shows how the addition of the covariance changes the statistical *t*-test and the importance of estimating the most appropriate one for each data set. Also note that *σ_tt’_* will assume different values at each time point when the correlation is not constant, for example, the structures UN and AR(1), but will assume exactly the same value in the case of CS. Remember that because the term *σ_tt’_* is dependent on *ρ*, when *ρ* goes to 0, *σ_tt’_* will also go to 0.

Now, let us consider that the variances among times are heterogeneous and the correlation between subjects is homogeneous (e.g., heterogeneous first-order autoregressive, ARH(1)) [6]. The standard error term to test differences between subjects at a given time *t* (*Y_it_ − Y_i’t_*), with *i* ≠ *i’*, is model 6:

*√* [2 * (*σ*^2^*_t_*)/(*r*)] (6)

and the error term to test differences within subjects at times *t* and *t’* given treatment *i* ≠ *i’* (*Y_it_ − Y_i’t’_*) is model 7

√ {2 * [(*σ*^2^*_t_* + *σ*^2^*_t’_*)/2]/*r*} (7)

where *σ*^2^*_t_* is the residual term variance at time *t*; *σ*^2^*_t’_* is the residual term variance at time *t’*; and *r* is the number of replicates.

Calculating the standard error term used to test differences in measurements taken on the same subject, such that *i* = *i’*, at times *t* and *t’*, such that *t* ≠ *t’* is more complex than those presented so far. First, we calculate a pooled variance between times *t* and *t’*; second, we subtract the covariance from the pooled variance term and then divide by the number of replicates according to model 8:

*√* (2 * {[(*σ*^2^*_t_* + *σ*^2^*_t’_*)/2] − *σ_tt’_*}/*r*) (8)

where *σ*^2^*_t_* is the variance of the residual term at time *t*, *σ*^2^*_t’_* is the variance of the residual term at time *t’*, *σ_tt’_* is the covariance for measurements taken at times *t* and *t’*, and *r* is the number of replicates.

The next sections of this paper provide examples of SAS programs to perform statistical analysis on annual (corn) and perennial (alfalfa) repeated measures data and show selected sections of the output files for discussion. The studies are described below. In this paper, we do not draw conclusions on corn grain yield or on alfalfa yield as a function of the treatments applied; rather, we demonstrate how to use PROC MIXED [9] to perform inference on these types of data sets.

## 4. Experimental Design Used for Data Collection on Examples Used in the Manuscript

This paper is divided into two sections and uses two different data sets to illustrate different methods of statistical analysis that would be appropriate for these data sets. The objective of the studies used as examples in this manuscript was to test the effects of a new fertilizer being produced at a turkey manure incinerator facility built in Minnesota as a source of phosphorus (P) and potassium (K) for crop production. The new fertilizer is referred to as TMA from hereon. The TMA was compared with triple superphosphate and potassium chloride, referred to as FERT, which are the most common sources of P and K used for crop production worldwide. The treatments consisted of two rates of TMA and FERT compared with an unfertilized control. Corn and alfalfa were the test crops because they were potential crops that would be fertilized with TMA in Minnesota. Both experiments were set up in a complete randomized block design with four blocks. There was no re-randomization of plots in consecutive years, such that the same plots always received the same treatments in all years. The corn study was repeated for three years and was replicated at only one location; more details of this study can be found in [10]. The alfalfa study was repeated for two years and replicated at the same three locations each year; more details of this study can be found in [11].

In the first section of this manuscript, we use the corn study to illustrate the effects of treating year as a fixed effect, a random effect, and a repeated measurement. The objective of this section is to show how the use of different approaches results in the calculation of different *F−* and *t*-tests, and how ignoring the presence of correlation can lead to the erroneous interpretation of results.

In the second section of this paper, we use the alfalfa study. In this section, year is considered only a repeated measurement, location is treated as a random effect, and treatment is treated as a fixed effect. The locations used in this study were all in Minnesota. The objectives of this section are to illustrate how to perform statistical analysis on data sets that have repeated measures combined with fixed and random effects.

This manuscript does not present any new mathematical models that can be used for statistical analysis but rather uses known models as an aid to help researchers choose the most appropriate approach for statistical analysis. This manuscript is not novel for researchers who have a greater knowledge of statistical analysis and keep up to date on new approaches for statistical analysis; however, graduate students and researchers who have less knowledge on this subject could greatly benefit from this manuscript.

## 5. Section I: Choosing the Best Approach for Statistical Analysis for the Corn Study

### 5.1. Visual Display

**Figure 1:**
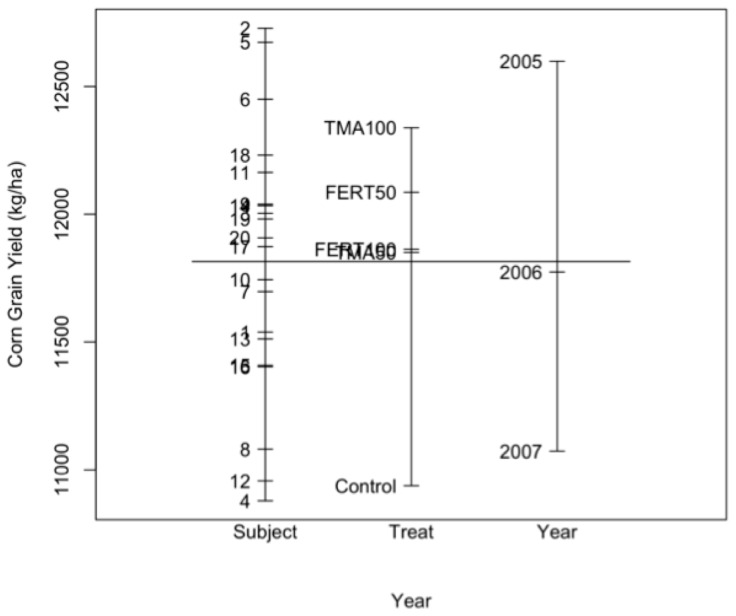
Plot of means for corn grain yield for the factors subject (plot (subject), averaged over year), treatment (Treat, averaged over year and block), and year (averaged over treatment and block).

Figure 1 shows a breakdown of means by the factors: plot, treatment, and year. The vertical line in the graph represents the overall mean corn grain yield. The horizontal lines, from left to right, are the means for each plot (averaged over year), treatment (averaged over plots and year), and year (averaged over plots and treatments). Figure 1 shows evidence for a possible significant treatment effect, as evidenced by the distance between the Control mean and other treatment means. When averaged over year, the treatment “Control” seems to have the lowest mean, and the treatment “TMA100” seems to have the highest. This figure also shows that when averaged over treatment, there is a trend of a decrease in corn grain yield from 2005 to 2007. 

### 5.2. Constructing the Statistical Models for the Corn Study

The first step in the statistical analysis of trials that contain mixed effects in the model is to construct a mathematical model that relates the variable response *Y* to all explanatory variables *μ* (effects) and contains a description of the random variation *ε* (error) affecting the observed response. For example, a simple model can be written as:

*Y*_*ij*_ = *μ*_*i*_ + *ε*_*ij*_ (9)

If we assume that *ε_ij_* is the random variation associated with the measurement of the *j*th subject response of the *i*th treatment, it is then independent identically distributed (*iid*) with a normal distribution (*N*), and then *Y_ij_* can be written as *Y_ij_ ~ iid N*(*μ_i_*, *σ*^2^*_Y_*). 

The components of the explanatory variables, *μ_i_*, and the source of variation, *ε_ij_*, will depend on the experimental design used to obtain *Y_ij_*. In the case of the corn study, we define our statistical model as:

*Y_ijt_* = *μ_it_* + *b_j_* + *ε_ijt_*,  *i* = 1, …, 5; *t* = 1, 2, 3; *j* = 1, …, 4. (10)

When time is considered a fixed effect, the variables present in Model (10) are defined as:

*μ_it_* = *μ..* + *α_i_* + *β_t_* + (*αβ*)*_it_* is the mean of treatment *i* at time *t*, with main effects for treatment (*α_i_*), time (*β_t_*), and their interaction ((*αβ*)*_it_*); *b_j_* is the random block effect; and *ε_ijt_* is the random error associated with the measurements taken at time *t* on the *j*th subject that was assigned to treatment *i*. As a result of the random block term in the model, the variance of *Y_ijt_* becomes Var[*Y_ijt_*] = *σ*^2^_Y_ = *σ*^2^_J_ + *σ*^2^, and the covariance of *Y_ijt_* is Cov[*Y_ijt_, Y_ijt’_*] = 0. The components *σ*^2^_j_ and *σ*^2^ are the variance terms for the block and residual terms, respectively, and are assumed to be independent of each other. This model has a multivariate normal distribution, *MVN(μ_it_*, *σ*^2^*_Y_*).

For the case when year is considered a random effect, Model (10) becomes Model (11):

*Y_ijt_* = *μ_i_* + *β_t_* + (*αβ*)*_it_* + *b_j_* + *ε_ijt_*,  *i* = 1, …, 5; *t* = 1, 2, 3; *j* = 1, …, 4. (11)

and is defined as:

*μ_i_ =μ.* + *α_i_* is the mean of treatment *i* with the main effect for treatment; *β_t_* is the random year effect; *αβ _it_* is the random interaction between the *i*th fixed treatment effect and the *t*th random year effect; *b_j_* is the random block effect; and *ε_ijt_* is the random error associated with the measurements taken at time *t* on the *j*th subject that was assigned to treatment *i*. Because year is a random term in the model, the interaction term treatment*year must also be considered a random term [12]. Therefore, the variance of *Y_ijt_* becomes Var[*Y_ijt_*] = *σ*^2^_Y_ = *σ*^2^_J_ + *σ*^2^_T_ + *σ*^2^_IT_ + *σ*^2^. All terms in *σ*^2^*_Y_* are considered to be independent of each other. This model also has a multivariate normal distribution, *MVN(μ_i_*, *σ*^2^*_Y_*), but remember that in this model, the structure of *σ*^2^*_Y_* differs from that of Model (10). 

In the analysis of variance model for repeated measures, we add a random term *C_ij_*, which represents the variation within subjects (in this case, in a given plot) as opposed to the error term *ε_ijt_*, which represents variation between subjects. As a result, Model (10) becomes Model (12):

*Y_ijt_* = *μ_it_* + *b_j_* + *C_ij_* + *ε_ijt_*,  *i* = 1, …, 5; *t* = 1, 2, 3; *j* = 1, …, 4. (12)

and is defined as:

*μ_it_* = *μ..* + *α_i_* + *β_t_* + (*αβ*)*_it_* is the mean of treatment *i* at time *t*, with main effects for treatment (*α_i_*), time (*β_t_*), and their interaction ((*αβ*)*_it_*); *b_j_* is the random block effect; *C_ij_* is the random effect of the *j*th subject submitted to the *i*th treatment; and *ε_ijt_* is the random error associated with the measurements taken at time *t* on the *j*th subject that was assigned to treatment *i*. The variance of *Y_ijt_* becomes Var[*Y_ijt_*] = *σ*^2^_Y_ = *σ*^2^_J_ + *σ*^2^, similar to the variance of *Y_ijt_* when year was a fixed effect, and the covariance of *Y_ijt_* is now Cov[*Y_ijt_, Y_ijt’_*] = *σ_tt’_*, for *t* ≠ *t’*. This model has a multivariate normal distribution, *MVN(μ_i_*, *σ*^2^*_Y_*).

A more simplified way to express (10–12) is by using matrix notation:

**Y = Xβ + Zu + ε** (13)

where **Xβ** represents an **X** matrix of the **β** fixed effects; **Zu** represents a **Z** matrix of **u** random effects; and **ε** represents the residual error. This model is discussed in more detail by [2,12] and many other statistics books that deal with mixed models.

### 5.3. Corn Analysis Results: Comparison of the Different Approaches 

#### 5.3.1. Year as a Fixed Effect

When treating year as a fixed or random effect, the first step is to check for homogeneity of variances between years, one of the assumptions for conducting any analysis of variance. If we assume that a data set has homogeneous variances, but in reality, it has heterogeneous variances, we will inflate the Type I error rate by computing incorrect residual variance components. Furthermore, it will also result in the estimation of incorrect standard errors used to test treatment differences, as illustrated in the previous sections. However, one can test the models by fitting the homogeneous variance model and heterogeneous variance model to the data. The choice of the best model is then based on indices of the goodness of fit, such as the Akaike’s information criterion (AIC) value, and the model with the lowest AIC value should be selected [13]. When the data from the corn study were tested with a model accounting for the heterogeneous variance among years, the analysis did not converge. Consequently, we assumed this data set to have homogeneous variances among years. The following Proc Mixed SAS program considers the data set to have homogeneous variances. In the case of heterogeneous variances, the statement repeated/group = year; is added to the program after the random__; statement. The program also performs a multiple comparison of least-squares means (lsmeans treat * year/diff;) and a specific contrast (estimate ‘Control vs. TMA’…) analysis that will be used for comparison purposes between approaches.

- proc mixed cl data = Corn;
- class block treat year;
- model yield = treat year treat * year/ddfm = kr;
- random block;
- lsmeans treat * year/diff;
- estimate ‘Control vs. TMA’
- treat 2 -1 -1 0;
- estimate ‘Control vs. Fert’
- treat 2 0 0 -1 -1;
- estimate ‘TMA vs. Fert’
- treat 0 1 1 -1 -1;
- estimate ‘50 vs. 100 kg P_2_O_5_ ha^−1^’
- treat 0 1 -1 1 -1;
- estimate ‘Control vs. All’
- treat 4 -1 -1 -1 -1;
- run;

Selected sections of the output are presented in Output 1 (Table 1). The “Covariance Parameter Estimates” section in Table 1 shows the estimate of the variances for the two random parameters listed in Model (10). The variance for the block term *b_j_* = 132,860, and the residual variance (*σ*^2^) or experimental error *ε_ijt_* = 592,634. The table also shows the 95% confidence limits for these two parameters. 

The “Fit Statistics” section in Table 1 shows values that are used to test the goodness of fit of the model used. As mentioned before, we use the AIC value to make a decision on which model to use, and, for this example, the AIC is 755.1.

The “Type 3 test of fixed effect” section in Table 1 shows the degrees of freedom of the fixed effects, Num DF (numerator degrees of freedom); the error term degrees of freedom used in the *F*-test, Den DF (denominator degrees of freedom); and also the *F*-test and *p*-value associated with the *F*-test. From this table, we see that the interaction treatment * year is not significant (*p*-value > 0.547), but highly significant are the main effects for treatment and year (*p*-value < 0.01). For field studies, researchers might want to adopt a *p*-value of 0.10 instead of 0.05, which is justifiable due to the large variability in the field; we also adopt this value throughout the manuscript. 

The “Estimate” section in Table 1 shows the treatment comparisons using the ESTIMATE statement (which are contrast analyses). The Estimate values are the differences between the treatments being compared in each contrast, associated with the respective standard error and degrees of freedom used to test that difference. Note the higher standard error used any time when the treatment “Control” was included in the comparison. As we suspected from the visual inspection of the data, the control is the treatment with the lowest mean and greater variability. The contrast analysis also shows that the other treatments have similar means. 

Even though there was not a significant treatment * year interaction, we show the “Differences of Least Square Means” table obtained by the “lsmeans treat * year/diff;” statement for comparison between the two approaches (year as a fixed effect and repeated measure). This section in Table 1 shows that one standard error (544.35) with the same degrees of freedom (42) was used to compare differences among all treatment means. The standard error was computed using Model (4); recall *√* [2 * (*σ*^2^)/(*r*)] = √ [2 * (592,634/4)] = 544.35.

#### 5.3.2. Year as a Random Effect

Now, the variable year is assumed to be a random effect. The following SAS program can be used to perform statistical analysis when year is a random effect:

- proc mixed cl data = Corn;
- class block treat year;
- model yield = treat;
- random block year year * treat;
- estimate ‘Control vs. TMA’
- treat 2 -1 -1 0;
- estimate ‘Control vs. Fert’
- treat 2 0 0 -1 -1;
- estimate ‘TMA vs. Fert’
- treat 0 1 1 -1 -1;
- estimate ‘50 vs. 100 kg P_2_O_5_ ha^−1^’
- treat 0 1 -1 1 -1;
- estimate ‘Control vs. All’
- treat 4 -1 -1 -1 -1;
- run;

Selected sections of the output are presented in Output 2 (Table 2). In Output 2, the “Covariance Parameter Estimates” section in Table 2 shows the estimate of the variances for the four random parameters listed in Model (11): *β_t_* =557,598, (*αβ*)*_it_* = −18,805, *b_j_* = 132,860, and *ε_ijt_* = 592,634.

The “Fit Statistics” section of Table 2 shows that the values used to test the goodness of fit of this model are much higher than those when year was a fixed effect. The AIC value here is 916.9. To compare these two models, we use a *χ*^2^ test by subtracting the AIC of the model with the greatest value minus the AIC of the model with the lowest value, 916.9 − 755.1 = 161.8. For degrees of freedom, we subtract the number of parameters from the model with the higher number minus the model with the lower number, 4 − 2 = 2. By checking the *χ*^2^ table at the *α*-value 0.10, we find 161.8 > 4.61 and accept that the model with the lowest AIC is the best model. 

When considering year as a random effect, the factor “year” cannot be included in the “model” statement in SAS; as a result, the main effect of “year” and the interaction “year * treat” cannot be tested by a multiple comparison test as was obtained when using the “lsmeans __/diff” statement. However, significant interactions between random effects can be tested by using the best linear unbiased predictor (BLUP) [2] approach, as demonstrated in the next section of this manuscript. For more detail on BLUP, the reader is referred to [2].

#### 5.3.3. Year as a Repeated Measure

As mentioned previously, when performing repeated measures analysis on a data set, it is important to select the covariance structure that best fits the model. The selection criteria used are the same as those just used to choose whether year is a fixed or random effect. For example, one would fit the model several times with possible covariance structure candidates and then select the one with the lowest AIC value. The following SAS code adds the first-order autoregressive, AR (1), structure, as this structure was found to be the best fit in terms of covariance structure. Recall that with this model, the variances among times are assumed to be homogeneous, and the correlation between subjects is heterogeneous for AR(1):

- proc mixed cl data = corn;
- class block treat year;
- model yield = treat year treat * year/ddfm =kr;
- random block;
- repeated/sub = block * treat type = AR(1) r rcorr;
- lsmeans treat|year/diff;
- estimate ‘Control vs. TMA’
- treat 2 -1 -1 0;
- estimate ‘Control vs. Fert’
- treat 2 0 0 -1 -1;
- estimate ‘TMA vs. Fert’
- treat 0 1 1 -1 -1;
- estimate ‘50 vs. 100 kg P_2_O_5_ ha^−1^’
- treat 0 1 -1 1 -1;
- estimate ‘Control vs. All’
- treat 4 -1 -1 -1 -1;
- run;

Selected sections of the output are presented in Output 3 (Table 3 and Table 4). Output 3 presents more information than Output 1 (Table 1). The “Estimated R Matrix for Subject 1” section of Table 3 shows the variance (*ε_ijt_* *=* *σ*^2^ = 620,963) in year one (Row1 Col1), year two (Row2 Col2), and year three (Row3 Col3). The values are all the same because the first-order autoregressive covariance structure assumes homogenous variances among years. The values of the diagonal are covariance terms from Model (12) *C_ij_* = Cov[*Y_it_, Y_it’_*] = *σ_tt’_*. We can see from this table that measurements taken one year apart have a covariance value of 248,471, while measurements taken two years apart have a covariance value of 99,423. The “Estimate R Correlation Matrix for Subject 1” section in Table 3 shows the correlation (*ρ*) for each pair of times (*t* and *t’*). The correlation is calculated based on the variance and covariance terms as *ρ* = *σ_tt’_/σ*^2^. In this output, we see that *ρ*^2−1^ = *σ_12′_/σ*^2^ = 248,471/620,963 = 0.4001; and *ρ*^3−1^ = *σ_13′_/σ*^2^ = 99,423/620,963 = 0.1601. Now, it is easy to see that the closer the correlation gets to 1 or −1, the higher the estimate of the covariance; the closer the correlation gets to 0, the lower the estimate of the covariance. Note that *ρ* can be seen as the percentage of the variance that was due to taking repeated measurements on the same plot. For example, a *ρ* of 0.40 means that 40% of the variance between years one unit apart from each other was due to variability within the plot. Therefore, when the data set has a very small or no correlation, treating year as a fixed effect or repeated measure will yield similar estimates of standard errors. However, when the correlation is not negligible, using too simple a model, such as the two methods presented before, can lead to erroneous inferences on treatment effects and inflate error terms, in this case by as much as 40%. Furthermore, the results from the contrast analysis in Output 1 (Table 1) show only one significant difference between treatments and years, whereas in Output 3 (Table 3), the same table now shows three significant differences (see Estimate section in Table 3). This is presented here to show the significance of using appropriate models and not to “search” for a model that will yield differences. Using the most appropriate model at once should lead the research to the right answers.

The “Covariance Parameter Estimates” section in Table 3 shows a summary of the previous two tables plus the random terms that are present in the current model. In Model (12), the only term that has not been reported yet is block (*b_j_*), which is found to be 94,701. By comparing Outputs 1, 2, and 3, one can see that the estimates of the block (132,860) and experimental error variances (592,634) are the same for the models that do not account for the correlation, Models (10) and (11). For Model (12), the block variance is smaller when compared with the other two models, and the experimental error is higher. This result shows that the addition of the correlation allowed for the estimation of more precise block and experimental variances. 

The “Fit Statistics” table shows that the values used to test the goodness of fit of this model are lower than those when year was either fixed and not correlated or a random effect. The AIC value here is 752.2. We use the *χ*^2^ test again to test Models (10) and (12), 755.1 − 752.2 = 2.9, with 1 degree of freedom. By checking the *χ*^2^ table at the *α*-value 0.10, we find 2.9 > 2.71, and we accept that the model with the lowest AIC is the best model.

From the table “Type 3 Tests of Fixed Effects”, we can see no significant interaction between treatment and year, but there are significant main effects. There are some remarkable differences among this table and those of Outputs 1 and 2. The main effect of “treatment” has a *p*-value of 0.001 for Model (10), 0.0124 for Model (11), and 0.039 for Model (12). The interaction treatment * year also has different *p*-values: for Model (10), it is 0.5466, and for Model (12), it is 0.3751. The degrees of freedom for all of the fixed effects are the same in Models (10) and (12), but the denominator degrees of freedom are not. The model with correlation was able to estimate degrees of freedom that are more realistic with the model [14].

By comparing the “Estimates” table among all outputs, we can see that all models compute the same “Estimate” value. The main differences among the three models are the standard errors and degrees of freedom associated with each standard error. Equation (12), (Output 3) has higher standard errors associated with each contrast analysis, which is a result of the higher estimated variance term. As pointed out before, the control treatment had the lowest mean and was more variable than the other treatments, while the fertilizer treatments did not differ from each other.

The “Differences of Least Square Means” table shows differences in the standard errors by treating year as a fixed effect or a repeated measure. In Output 1, there was only one standard error used to compare any treatment mean for any year. However, in Output 3, we can see that there are three different standard errors. Those standard errors were calculated based on Models (4) and (5). The standard error term to test for differences among treatments in any year, for all *i ≠ i’*, was computed as √ 2 * (620,963/4) = 557.21; to test differences in the same treatment between years one unit apart, it was computed as √ [2 * (620,963 − 248,471)/4)] = 439.08, and for years two units apart, it was computed as √ [2 * (620,963 − 99,423)/4)] = 523.93. 

The major difference between Output 1 and Output 3 is that the standard error and degrees of freedom used to test the same treatment with means one or two years apart were lower in the model that included the covariance parameter in the calculations, whereas when different treatments, such that *i ≠ i’*, were compared in any year, the model with correlation added had a higher standard error. This shows how important it is to choose the covariance structure that best fits the data set. In addition, Power calculations using Model (10) showed a Power value for the interaction treat * year of 0.34903, while for Model (12), the value of the Power was improved to 0.42341. This difference represents an improvement in the Power to detect a significant *F*-test (*α* < 0.05) of 21%. 

All of the results presented in this section show that adding a correlation to the model resulted in an improved statistical analysis, even though the *p*-values did not change much in this example. Larger differences in *p*-values will likely happen in many cases when the covariance between measurements taken on the same subject over time is large. Therefore, the repeated measures model approach should be used instead of the fixed model approach.

## 6. Section II: Constructing the Statistical Models for the Alfalfa Study

In the alfalfa yield trial, we add another source of variation to the model. We include location as a variable (*ι_l_*). The locations and soil series used in this study represented locations and soils where TMA would likely be applied; hence, this effect is most appropriately treated as a random effect in our model. However, by making such an assumption, it is also implied that treatment and year effects at specific locations are not important. In most field trials, if not all, this assumption is false. Therefore, we must verify whether the treatments behaved in a similar way at all locations, whether the locations behaved similarly in all years, and whether treatments behaved similarly at all locations in all years and should be treated as a fixed effect. Because the “location” (*ι_l_*) effect is random, the interaction terms “treatment × location” (*αι*)*_il_*, “year × location” (*βι*)*_tl_*, and “treatment × location × year” (*αβι*)*_ikl_* are also random terms. For reasons of space, we only model the alfalfa study as a repeated measures data set. The model for the alfalfa trial can be defined as:

*Y*_*ijtl*_ = *μ*_*it*_ + *C*_*ij*_ + *ι*_*l*_ + *b(ι)*_*jl*_ + (*τι*)_*il*_ + (*βι*)_*tl*_ + (*αβι*)_*itl*_ + *ε*_*ijtl*_ (14)

with *i* = 1, …, 5; *j* = 1, …, 4; *t* = 1, 2; *l* = 1, 2, 3,

where *μ_it_ =* *μ..* + *α_i_* + *β_t_* + (*αβ*)*_it_* denotes the intercept, the fixed effects of treatment, year, and their interaction; *C_ij_* + *ι_l_* + *b*(*ι*)*_jl_* + (*αι*)*_il_* + (*βι*)*_tl_* + *(αβι)_ikl_* + *ε_ijtl_* denotes the random effect of the *j*th subject submitted to the *i*th treatment, the *j*th block effect at the *l*th location, the *il*th treatment × location interaction effect, the *tl*th year × location interaction effect, the *itl*th treatment × location × year interaction effect, and the overall error term *ε_ijtl_*, respectively. The variance of *Y_ijtl_* is, therefore, Var[*Y_ijtl_*] = *σ*^2^_Y_ = *σ*^2^*_L_*, *σ*^2^*_IL_*, *σ*^2^*_TL_*, *σ*^2^*_ITL_*, *σ*^2^, and the covariance of *Y_ijtl_* is Cov[*Y_ijlt_, Y_ijlt’_*] = *σ_tt’_*, for *t* ≠ *t’*, and again, the most appropriate form of *σ_tt’_* must be estimated as described in the previous section. This model has a multivariate normal distribution, *MVN(μ_i_*, *σ*^2^*_Y_*).

### 6.1. Visual Display

**Figure 2:**
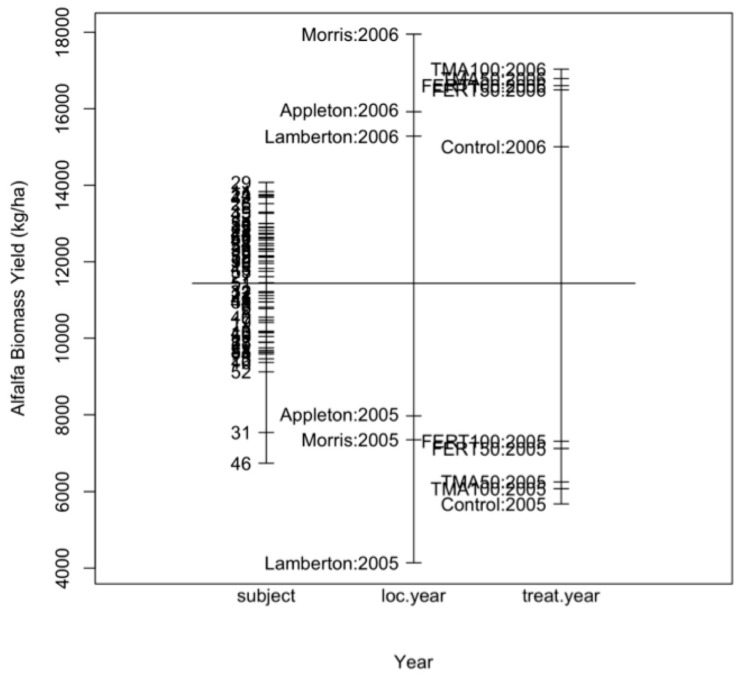
Plot of means of alfalfa biomass yield for the factors subject (plot (subject), averaged over location and year), location in each year (averaged over treatment and block), and treatment in each year (averaged over location and block).

Figure 2 shows a breakdown of means by the factors: plot, location in each year, and treatment in each year. The vertical line in the graph represents the overall mean of alfalfa biomass yield. The horizontal lines, from left to right, are the means for each plot (averaged over year and location), location in each year (averaged over plots and treatment), and treatment in each year (averaged over plots and locations). This figure indicates a possible significant location-by-year interaction effect; in addition, it shows evidence of a treatment-by-year interaction effect. In 2005, the treatments Control, TMA50, and TMA100 seem to be the lowest, while FERT50 and FERT 100 seem to be the highest. In 2006, however, the treatment Control seems to have the lowest yield, while the other four treatments seem to have similar mean yields. 

### 6.2. Inference on Random Effect Interactions

Statistical analysis on data sets that have a large number of parameters included in the model can be challenging, and caution must be taken so that the researcher does not specify too many parameters. The statistical model for the alfalfa study is an example of such a complex model. It includes fixed effects, repeated measures, and random effect interactions that might have importance from an agronomic standpoint. As a result, the main effect of location and all of its interactions cannot be included in the MODEL statement together with the fixed effects in SAS Proc Mixed programs. They need to be specified in the RANDOM statement. Therefore, differences among treatments at specific locations (treatment-by-location interaction) are better assessed by location-specific inference using BLUP, as described by [2]. 

Differences among locations in a specific year (year * location interaction) and treatment by specific location and year (treatment * location * year interaction) can be assessed with a similar methodology to that for treatment at a specific location, as mentioned above. This analysis, however, must be performed in a series of steps, which is discussed in the following section. As in the previous section, we mainly present SAS programs that can be used to perform the desired tests. In addition, this section only presents results from a statistical analysis that considers year to be a repeated measurement and treatments analyzed within a year. 

### 6.3. Assessing Main Effects and Interaction to Be Tested

The visual analysis of the data showed very little evidence for a significant three-way interaction among treatment, location, and year. However, we can still check the value of this variance term and compare it with the residual variance to assess how large it really is before making any inference. We ran the following program to estimate the random terms in our model (this initial model does not add any correlation).

- proc mixed cl data = Alfalfa_RMA nobound;
- class site block treat year;
- model yield = treat year treat * year;
- random site block(site) site*year site*treat site * treat * year;
- run;

Output 4 (Table 5) shows a selected part of this output. The table ‘Covariance Parameter Estimates’ shows that the variances for interactions ‘loc by treat’ and ‘loc by treat by year’ are much smaller than the variance of the residual term. Based on this result, we accept that no three-way interaction is present. This table also shows that the interaction location by year is larger than the residual variance. Now, we have enough evidence to look for a possible interaction suggesting that locations behaved differently in each year and illustrate how to check for this interaction in three steps.

#### 6.3.1. Step One

First, due to the fact that location is considered random *iid N*(*0*, *σ*^2^), we need to specify a linear combination of fixed and random effects that explains the difference between two locations *ι* and *ι’* at a given time *t*. Differences in BLUPs can be computed as:

*ι*_l_ − *ι*_l’_ + (*βι*)*_lt_* − (*βι*)*_l’t’_*, for all *l* ≠ *l’*, and *t* = *t’* (15)

where *ι_l_* is the effect of the *l*th location, and (*βι*)*_lt_* is the *lj*th location × year interaction effect. 

The location effect in each year is tested by simultaneous contrasts, such as

*ι*_1_ − *ι*_2_ + (*βι*)_11_ − (*βι*)_21_ (16)

*ι*_1_ − *ι*_3_ + (*βι*)_11_ − (*βι*)_31_ (17)

*ι*_2_ − *ι*_3_ + (*βι*)_21_ − (*βι*)_31_ (18)

Note that this procedure must be performed for each year separately. The following SAS program was used to test the location effect in a specific year:

- proc mixed cl data = alfalfa_study;
- class rep loc treat year;
- model yield = treat year treat * year/ddfm = KR;
- random loc rep(loc) loc * treat loc * year;
- repeated year/sub = rep * treat * loc type = un R Rcorr;
- contrast ‘Location effect at Year 1’
- |loc 1 −1 0
- loc * year 1 0 −1 0,
- |loc 0 1 −1
- loc * year 0 0 1 0 −1 0,
- |loc 1 0 −1
- loc * year 1 0 0 0 −1 0;
- contrast ‘Location effect at Year 2’
- |loc 1 −1 0
- loc * year 0 1 0 −1,
- |loc 0 1 −1
- loc * year 0 0 0 1 0 −1,
- |loc 1 0 −1
- loc * year 0 1 0 0 0 −1;
- Run;

Output 5 (Table 6) shows the “Contrast” table, where we can see a significant location effect in both years (*p*-values < 0.001). However, so far, we do not have any evidence for a location-by-year interaction effect. Note that if the *p*-values resulting from the contrast analysis for both years were > 0.05, then the BLUPs at the three locations would be statistically the same; a *p*-value < 0.05 in one year and >0.05 in the second indicate a significant interaction between location and year; and if the *p*-values are < 0.05 in both years, it indicates that there are locations that behaved differently in each year but does not tell us specifically what happened. In the alfalfa study, we see that the *p*-values were < 0.05; therefore, we must proceed to step two.

#### 6.3.2. Step Two

Based on the results from the first step, we need to investigate further to determine if there was a significant interaction between location and year or if the significant differences that we see in Output 5 were the same for both years. In other words, location 1 could have been higher than locations 2 and 3 for both years, and no interaction would be present. Alternatively, different locations had the highest mean in different years, which would indicate an interaction. In the second step, an unbiased pre-planned comparison based on location-specific characteristics is computed. In the alfalfa study, two locations, one and three, had a more acidic soil pH, around 5.5, while in location two, the soil pH was near neutral, 6.5. Because alfalfa yield is expected to be higher at a soil pH of around 6.8, we can base our comparison on the different soil pH levels.

Because we can only make two contrast analyses in this study, due to the number of degrees of freedom for location (3 − 1 = 2), it makes more sense to compare the BLUPs from location 1 against 3 and from 2 against 3 for both years. The difference in BLUP between two locations in years one and two are computed as follows:

Year 1

*ι*_1_ − *ι*_2_ + (*βι*)_11_ − (*βι*)_21_ (19)

*ι*_1_ − *ι*_3_ + (*βι*)_11_ − (*βι*)_31_ (20)

*ι*_2_ − *ι*_3_ + (*βι*)_21_ − (*βι*)_31_ (21)

Year 2

*ι*_1_ − *ι*_2_ + (*αι*)_12_ − (*αι*)_22_ (22)

*ι*_1_ − *ι*_3_ + (*αι*)_12_ − (*αι*)_32_ (23)

*ι*_2_ − *ι*_3_ + (*αι*)_22_ − (*αι*)_32_ (24)

To compute the BLUP for each location in each year, the following function can be used:

*μ* + *ι _lt_* + (1/*y*) *Σ_t_ β_t_* + (1/*y*) *Σ_t_* (*βι*)*_lt_* (25)

where *μ* denotes the intercept; *ι _lt_* is the effect of the *l*th location; (1/*y*) *Σ_t_ β_t_* is the averaged mean of all years at the *l*th location, with *y* being the number of years; and (1/*y*) *Σ_t_* (*βι*)*_lt_* is the *lt*th location × year interaction effect.

The following SAS “ESTIMATE” statements can be added to the previous SAS program to compute the BLUP for each location in each year and the specific difference between those BLUPs:

estimate ‘Location 1 Year 1’

- intercept 1
- year 1 0
- |loc 1 0 0
- loc * year 1 0;

estimate ‘Location 3 Year 2’

- intercept 1
- year 0 1
- |loc 0 0 1
- loc * year 0 0 0 0 0 1;

estimate ‘Location 1 vs. Location 3 Year 1’

- | loc 1 −1 0
- loc * year 1 0 0 0 −1 0;

estimate ‘Location 2 vs. Location 3 Year 1’

- | loc 0 1 −1
- loc * year 0 0 1 0 −1 0;

estimate ‘Location 1 vs. Location 3 Year 2’

- | loc 1 0 −1
- loc * year 0 1 0 0 0 −1;

estimate ‘Location 2 vs. Location 3 Year 2’

- | loc 0 1 −1
- loc * year 0 0 0 1 0 −1;

#### 6.3.3. Step Three

The third and last step consists of examining the *p*-values obtained by running the above ESTIMATE statements. Output 6 (Table 7) shows the “Estimate” table. The data show that Loc 1 vs. Loc 3 was significantly different in year 1 but was not different in year 2. Loc 2 vs. Loc 3 was statistically the same in year 1, but in year 2, they were statistically different. Therefore, we can conclude that there was a significant location-by-year interaction effect. The following “Estimate” section in Table 7 shows the BLUPs from each location in each year, which helps to visualize and explain the significant location-by-year interaction. Locations 1 and 2 were statistically different in year one but were statistically the same in year two. This can probably be attributed to the different soil pH and establishment year effects on alfalfa yield. In addition, the table “Type 3 Tests of Fixed Effects” section shows that there is a significant treatment-by-year interaction, as suggested by Figure 2.

## 7. Conclusions

Treating data sets collected over years on agronomic field trials as repeated measures data was shown to have more power to detect treatment differences than the approaches conventionally (univariate or multivariate analysis) used, such as simple ANOVA or regression models. This was mainly due to the addition of a covariance structure to measurements taken at continuous time periods on the same subject. Even though repeated measures analysis was shown to be the best approach for these data sets, a detailed examination of the data must be performed before final inferences on treatment effects can be made. The correct covariance model must be fit so the correct correlation can be estimated and correct *F−* and *t*-tests can be computed. Statistical analysis of mixed model data sets, such as those in the alfalfa study, requires more interaction between the software and researcher so that precise inference on treatment effects can be computed. 

## 8. Contributions and Future Work Needed

This manuscript contributes essential statistical tools that can be used by researchers conducting field research on perennial and annual crops. These types of research are highly correlated, and mishandling of the correlation between sample collections can lead the researcher to make erroneous inferences on the real effect of the treatments being tested. Future work should assess whether the methods provided in this manuscript fit all cases, or if adaptions are needed for site-specific issues.

## Figures and Tables

**Table 1:** Output 1: Partial output of the statistical analysis of the corn study with year treated as a fixed effect (homogeneous).

Covariance Parameter Estimates							
Cov Parm Group		Estimate	Alpha	Lower		Upper	
block		132,860	0.05	34,207		7,832,096	
Residual		592,634	0.05	402,912		957,381	
		Fit Statistics					
		−2 Res Log Likelihood		751.1			
		AIC (smaller is better)		755.1			
		AICC (smaller is better)		755.4			
		BIC (smaller is better)		753.8			
Type 3 Tests of Fixed Effects							
		Num	Den	F Value		Pr > F	
Effect		DF	DF				
treat		4	42	5.68		0.001	
year		2	42	19.69		<0.0001	
treat * year		8	42	0.87		0.5466	
Estimates							
Label		Estimate	Standard Error	DF	t Value	Pr > |t|	
Control vs. TMA		−2314.83	544.35	42	−4.25	0.0001	
Control vs. Fert		−2074.5	544.35	42	−3.81	0.0004	
TMA vs. Fert		240.33	444.46	42	0.54	0.5915	
50 vs. 100 kg P_2_O_5_ ha^−1^		−264.83	444.46	42	−0.6	0.5545	
Control vs. All		−4389.33	993.84	42	−4.42	<0.0001	
Differences of Least Square Means							
Effect	t yr	_t_yr	Estimate	Standard Error	DF	t Value	Pr > |t|
treat * year	1 1	1 2	835.5	544.35	42	1.53	0.1323
treat * year	1 1	1 3	1947	544.35	42	3.58	0.0009
treat * year	1 1	2 1	−344.25	544.35	42	−0.63	0.5305
treat * year	1 1	2 2	−23.5	544.35	42	−0.04	0.9658
treat * year	1 1	2 3	409.25	544.35	42	0.75	0.4564

**Table 2:** Output 2: Partial output of the statistical analysis of the corn study when year was treated as a random effect.

Covariance Parameter Estimates						
Cov Parm	Estimate	Alpha	Lower	Upper		
block	132,860	0.05	−143,500	409,219		
year	557,598	0.05	−586,261	1,701,457		
treat * year	−18,805	0.05	−160,525	122,916		
Residual	592,634	0.05	402,912	957,381		
	Fit Statistics					
	−2 Res Log Likelihood		908.9			
	AIC (smaller is better)		916.9			
	AICC (smaller is better)		917.7			
	BIC (smaller is better)		914.4			
Type 3 Tests of Fixed Effects						
Effect	Num	Den	F Value	Pr > F		
	DF	DF				
treat	4	8	6.5	0.0124		
Estimates						
Label	Estimate	Standard Error	DF	t Value	Pr > |t|	
Control vs. TMA	−2314.83	508.63	8	−4.55	0.0019	
Control vs. Fert	−2074.5	508.63	8	−4.08	0.0035	
TMA vs. Fert	240.33	415.3	8	0.58	0.5787	
50 vs. 100 kg P_2_O_5_ ha^−1^	−264.83	415.3	8	−0.64	0.5415	
Control vs. All	−4389.33	928.63	8	−4.73	0.0015	

**Table 3:** Output 3: Partial output of the statistical analysis for the corn study with year treated as a repeated measure.

Estimated R Matrix for Subject 1					
Row	Col1	Col2		Col3	
1	620,963	248,471		99,423	
2	248,471	620,963		248,471	
3	99,423	248,471		620,963	
Estimated R Correlation Matrix for Subject 1					
Row	Col1	Col2		Col3	
1	1	0.4001		0.1601	
2	0.4001	1		0.4001	
3	0.1601	0.4001		1	
Covariance Parameter Estimates					
Cov Parm Subject	Estimate	Alpha	Lower	Upper	
block	94,701	0.05	18,728	1.06 × 10^8^	
AR(1) treat * year	0.4001	0.05	0.07588	0.7244	
Residual	620,963	0.05	399,907	1,093,942	
	Fit Statistics				
	−2 Res Log Likelihood	746.2			
	AIC (smaller is better)	752.2			
	AICC (smaller is better)	752.8			
	BIC (smaller is better)	750.3			
Type 3 Tests of Fixed Effects					
Effect	Num	Den	F Value	Pr > F	
	DF	DF			
treat	4	13.6	3.42	0.0388	
year	2	30	21.2	<0.0001	
treat * year	8	30.3	1.12	0.3751	
Estimates					
	Estimate	Standard Error	DF		Pr > |t|
				t Value	
Control vs. TMA	−2314.83	700.99	13.6	−3.3	0.0054
Control vs. Fert	−2074.5	700.99	13.6	−2.96	0.0106
TMA vs. Fert	240.33	572.35	13.6	0.42	0.6811
50 vs. 100 kg P_2_O_5_ ha^−1^	−264.83	572.35	13.6	−0.46	0.6509
Control vs. All	−4389.33	1279.82	13.6	−3.43	0.0042

**Table 4:** Output 3: Partial output of the statistical analysis for the corn study with year treated as a repeated measure-continued.

Differences of Least Square Means							
Effect	t yr	_t_yr	Estimate	Standard Error	DF	t Value	Pr > |t|
treat * year	1 1	1 2	835.5	439.08	28.7	1.9	0.0671
treat * year	1 1	1 3	1947	523.93	41.2	3.72	0.0006
treat * year	1 1	2 1	−344.25	557.21	31.3	−0.62	0.5412
treat * year	1 1	2 2	−23.5	557.21	31.3	−0.04	0.9666
treat * year	1 1	2 3	409.25	557.21	31.3	0.73	0.4681

**Table 5:** Output 4: Partial output of the statistical analysis for location (loc) as random effect in the alfalfa study, no correlation added.

Covariance Parameter Estimates				
Cov Parm Group	Estimate	Alpha	Lower	Upper
loc	1,583,509	0.05	−3,242,629	6,409,646
block (loc)	163,907	0.05	−98,528	426,342
Loc * year	1,383,329	0.05	−1,476,742	4,243,401
Loc * treat	−11,951	0.05	−265,153	241,251
loc * treat * year	83,594	0.05	−296,906	464,093
Residual	1,174,233	0.05	882,445	1,639,922

**Table 6:** Output 5: Partial output of the statistical analysis on the location-by-year interaction effect for the alfalfa study.

	Fit Statistics				
	−2 Res Log Likelihood		1889.8		
	AIC (smaller is better)		1903.8		
	AICC (smaller is better)		1904.9		
	BIC (smaller is better)		1897.5		
Type 3 Tests of Fixed Effects					
Effect	Num	Den	F Value	Pr > F	
	DF	DF			
treat	4	9.14	4.84	0.0226	
year	1	2	99.76	0.0099	
treat * year	4	53	2.78	0.0360	
Contrasts					
Label	Num	Den	F Value	Pr > F	
	DF	DF			
Location effect at year 1	2	25.2	49.7	<0.0001	
Location effect at year 2	2	58.7	13.3	<0.0001	

**Table 7:** Output 6: Partial output file of the statistical analysis on the location-by-year interaction effect for the alfalfa study.

Estimate					
		Standard			
Label	Estimate	Error	DF	t Value	Pr > |t|
Loc 1 vs. Loc 3 Year 1	−3122	397.57	13.8	−7.85	0.0001
Loc 2 vs. Loc 3 Year 1	−606	397.57	13.8	−1.52	0.1500
Loc 1 vs. Loc 3 Year 2	−657	516.66	31.7	−1.27	0.2127
Loc 2 vs. Loc 3 Year 2	1918	516.66	31.7	3.71	0.0008
Estimate					
		Standard			
Label	Estimate	Error	DF	t Value	Pr > |t|
Location 1 year 1	4203	281.85	13.6	14.91	<0.0001
Location 1 year 2	15,308	367.82	30.9	41.62	<0.0001
Location 2 year 1	7325	281.85	13.6	25.99	<0.0001
Location 2 year 2	17,883	367.82	30.9	48.62	<0.0001
Location 3 year 1	7931	281.85	13.6	28.14	<0.0001
Location 3 year 2	15,965	367.82	30.9	43.4	<0.0001

## Data Availability

Not applicable.
